# *Bacteriocins:* An Overview of Antimicrobial, Toxicity, and Biosafety Assessment by *in vivo* Models

**DOI:** 10.3389/fmicb.2021.630695

**Published:** 2021-04-15

**Authors:** Diego Francisco Benítez-Chao, Angel León-Buitimea, Jordy Alexis Lerma-Escalera, José Rubén Morones-Ramírez

**Affiliations:** ^1^Facultad de Ciencias Químicas, Universidad Autónoma de Nuevo León, San Nicolás de los Garza, Mexico; ^2^Centro de Investigación en Biotecnología y Nanotecnología, Facultad de Ciencias Químicas, Parque de Investigación e Innovación Tecnológica, Universidad Autónoma de Nuevo León, Apodaca, Mexico

**Keywords:** bacteriocins, antibiotics, antimicrobial resistance, antibacterial peptide, toxicity, biosafety, *in vivo* model

## Abstract

The world is facing a significant increase in infections caused by drug-resistant infectious agents. In response, various strategies have been recently explored to treat them, including the development of bacteriocins. Bacteriocins are a group of antimicrobial peptides produced by bacteria, capable of controlling clinically relevant susceptible and drug-resistant bacteria. Bacteriocins have been studied to be able to modify and improve their physicochemical properties, pharmacological effects, and biosafety. This manuscript focuses on the research being developed on the biosafety of bacteriocins, which is a topic that has not been addressed extensively in previous reviews. This work discusses the studies that have tested the effect of bacteriocins against pathogens and assess their toxicity using *in vivo* models, including murine and other alternative animal models. Thus, this work concludes the urgency to increase and advance the *in vivo* models that both assess the efficacy of bacteriocins as antimicrobial agents and evaluate possible toxicity and side effects, which are key factors to determine their success as potential therapeutic agents in the fight against infections caused by multidrug-resistant microorganisms.

## Introduction

According to the WHO, diseases caused by multidrug-resistant (MDR) pathogens are a serious worldwide public health problem (World Health Organization, 2019c). The rapid spread of MDR pathogens have reduced the effectiveness of common antibiotics (Gupta and Datta, 2019). Therefore, there is a particular need for the development of new antimicrobial agents, specifically those directed against MDR bacteria (Fair and Tor, 2014). Bacteriocins represent the most important group of antimicrobial peptides with applications in human health (Marshall and Arenas, 2003). The ability of bacteriocins to kill or inhibit relevant pathogenic bacteria (including MDR pathogens) *in vitro* has been well documented (Cui et al., 2012; Gabrielsen et al., 2014; Perez et al., 2014; Newstead et al., 2020). However, the use of animal models is an important part in the research toward the development of new therapeutic agents. There are many animal models used for screening drugs or chemical compounds in preclinical studies (Zwierzyna and Overington, 2017). Mice are the best-known animal models for *in vivo* bacteriocin efficacy studies. Other animal species, less frequently used, are guinea pigs, rabbits, and hamsters (Badyal and Desai, 2014). Therefore, the present review addresses the antimicrobial effects, toxicity, and biosafety of bacteriocins in *in vivo* systems. The bacteriocins here included are those naturally synthesized by native or recombinant producers, those chemically synthetized or bioengineered bacteriocins, those obtained from direct application of cultures with bacteriocin-producers, and those bacteriocins produced in cell-free supernatants (CFSs). Moreover, we here discuss the toxicity and biosafety *in vivo* assays, in different animal models, that have been reported during the exploration of antimicrobial effects.

## Antimicrobial Resistance: Global Emergency

The death toll caused by drug-resistant infections has been dangerously rising every year within the last two decades. According to World Health Organization (2019c), at least 700,000 people died annually around the world because of this. In Europe, deaths raised from 25,000 in 2007 (European Centre for Disease Prevention and Control, 2009) to 33,110 in 2015 (Cassini et al., 2019). Moreover, according to WHO Africa, 54,000 cases of MDR tuberculosis were detected in 42 countries, and approximately 3200 cases of extensively drug-resistant (XDR) tuberculosis were notified in eight countries from the African region from 2004 to 2011 (Ndihokubwayo et al., 2013). China, one of the most populated countries, reported that between 2005 and 2017, the range of isolated drug-resistant bacteria varied from 22,774 to 190,610 (Qu et al., 2019). During 2017 in India, a study reported that 70% of drug-resistant Gram-negative isolates corresponded to *Escherichia coli, Klebsiella pneumoniae, Acinetobacter baumannii*, and *Pseudomonas aeruginosa* (Taneja and Sharma, 2019). Regarding the United States, 23,000 deaths were counted in 2013; 6 years later, in 2019, numbers increased to 35,000 deaths associated with antibiotic-resistant infections (Frieden, 2013). In particular, Mexico has a dramatic lack of information and epidemiological surveillance for antibiotic resistance (Amabile-Cuevas, 2010). Recently, a 6-month study assessed the resistance rates of several bacterial pathogens in 47 Mexican centers. They included almost 23,000 strains, and their results showed that the most common drug-resistant strains were *E. coli*, *Klebsiella* sp., *Enterobacter* sp., *P. aeruginosa*, *Acinetobacter* sp., vancomycin-resistant *Enterococcus faecium*, and methicillin-resistant *Staphylococcus aureus* (MRSA) (Garza-González et al., 2019). According to the WHO, if severe actions are not taken by 2050, it is estimated that 10 million people will die annually around the world due to diseases caused by MDR pathogens. This situation may cause a severe economic impact with consequences similar to the 2009 Global Financial Crisis (World Health Organization, 2019c).

## Current Treatments for Bacterial Infections

Since their discovery in 1928, antibiotics have effectively controlled bacterial infections (Podolsky, 2018). The current treatments for bacterial infections include bactericidal or bacteriostatic agents from different classes of antibiotics. The most common classes of drugs are penicillins, cephalosporins, quinolones, macrolides, tetracyclines, glycopeptides, and monobactams, among others (Taylor et al., 2002).

Within the last two decades, a variety of antibiotics have been approved to treat infections caused by Gram-positive and Gram-negative bacteria. Novel antibiotics against Gram-positive bacteria include β-lactams (ceftaroline and ceftobiprole), glycopeptides (dalbavancin, oritavancin, and telavancin), oxazolidinones (tedizolid phosphate), quinolones (besifloxacin, delafloxacin, and ozenoxacin), and tetracyclines (omadacycline). The main advantage of the previously mentioned antibiotics is their efficiency in the treatment of bacterial infections caused by MDR strains (Koulenti et al., 2019b). Recently approved treatments to combat MDR Gram-negative bacteria include antibiotic combinations of β-lactam/β-lactamase inhibitors (ceftolozane/tazobactam, ceftazidime/avibactam, and meropenem/vaborbactam), and aminoglycosides (plazomicin) combined with tetracyclines (eravacycline) (Koulenti et al., 2019a). Nonetheless, misuse and overuse of these combinatorial treatments have contributed to further development of antibiotic resistance (Malik and Bhattacharyya, 2019).

The rapid spread of MDR and XDR bacteria in both hospitals and community settings has reduced the effectiveness of antibiotics (Gupta and Datta, 2019). One of the main concerns regarding antibiotic resistance is that some bacteria have become resistant to almost all currently available antibiotics. Therefore, these bacteria represent a severe public health problem worldwide. Of particular interest are the following strains: MRSA, vancomycin-intermediate and -resistant; *E. faecium*, vancomycin-resistant; *Enterobacteriaceae*, carbapenem-resistant, extended-spectrum beta-lactamase (ESBL)-producing; *A. baumannii*, carbapenem-resistant; and *P. aeruginosa*, carbapenem-resistant (World Health Organization, 2017).

Preventing and controlling the spread of antibiotic resistance is necessary to invest in research and development of new agents with therapeutic potential to treat bacterial infections. According to data from the WHO, there is an arsenal of 50 antimicrobial agents and their combinations in clinical development. Thirty-two are antibiotics active against the WHO priority pathogens, ten are biological agents, two are classified as innovative agents, and two are active against MDR Gram-negative bacteria (World Health Organization, 2019a).

## Bacteriocins as Alternative Antimicrobial Agents

There is a particular need for new antimicrobial agents, specifically those directed against antibiotic-resistant bacteria (Fair and Tor, 2014). Defensins and bacteriocins represent the most important groups of antimicrobial peptides with applications in human health (Marshall and Arenas, 2003). Defensins are small cysteine-rich (forming three to six disulfide bonds) cationic antimicrobial peptides ubiquitous among eukaryotes that form an essential element of innate immunity. They consist of two analogous superfamilies and an extensive convergent evolution is the source of their similarities (Shafee et al., 2016). Bacteriocins are ribosomally synthesized peptides secreted by a variety of bacteria for the purpose of killing other bacteria. Thereby, whereas defensins are important components of the host immune response against infection in eukaryotes (Arnett and Seveau, 2011), bacteriocins participate in removing microbial competition in prokaryotes (Cotter et al., 2013).

The way to classify defensins and bacteriocins has been based on their biochemical (net charge) and/or structural features (linear/circular/amino acid composition) and there is a current search for common patterns that might help to distinguish them (Tossi and Sandri, 2002; Zasloff, 2002). To determine whether all molecules are homologous or have independently evolved similar features, the best evidence lies in the structure. The antimicrobial peptides can be differentiated from their overall three-dimensional structure and the spacing of half-cystine residues involved in intrachain disulfide bonds (Marshall and Arenas, 2003). To date, only two bacteriocins (bactofencin and laterosporulin) have been expressed as defensin-like bacteriocins. Bactofencin is a disulfide bond-containing bacteriocin with highly conserved cysteine residues and structurally related to eukaryotic defensins due to their highly cationic nature (O’Shea et al., 2013; O’Connor et al., 2018). The laterosporulin has been previously identified from a *Brevibacillus laterosporus* strain GI-9 and contain disulfide bonds in positions homologous to eukaryotic defensins (Singh et al., 2012). The presence of disulfide connectivity suggests its similarity to β-defensins while its architectural similarity is related to α-defensins (Singh et al., 2015). Recently, laterosporuli10, a novel defensin-like bacteriocin (class II bacteriocin) from the *Brevibacillus* sp. strain SKDU10, was characterized. This bacteriocin showed 57.6% homology with laterosporulin and differences in the molecular weight and the number of cationic amino acids. It was highly efficient in killing *S. aureus* (Gram-positive bacteria) and the *Mycobacterium tuberculosis* H37Rv strain when compared to laterosporulin (Baindara et al., 2017). Interestingly, Class II bacteriocins are easily manipulated by genetic engineering techniques because they do not have large post-translational modifications and it is possible to obtain variants according to the technological requirements and needs (Zheng and Sonomoto, 2018; Kumariya et al., 2019). Therefore, this technique could be useful to develop new antimicrobial peptides to be used as an alternative to common antibiotics.

As stated above, bacteriocins represent an interesting solution to reduce the development of resistance. Besides, bacteriocins are continuously evolving with high potential against clinically relevant pathogens (Piper et al., 2009; Bonhi and Imran, 2019). Bacteriocins can be easily manipulated by bioengineering techniques (Field et al., 2010). Unlikely to antibiotics, bacteriocins can be engineered to attach anywhere on the cellular outer membrane because they do not have a specific receptor (Bonhi and Imran, 2019) and they can be produced *in situ* by probiotics (Dobson et al., 2012; O’Shea et al., 2012). Consequently, this represents a new path in bacteriocin research that will undoubtedly lead to the development of new therapeutic strategies with highly relevant clinical applications (Chikindas et al., 2018).

## Antibacterial Activity of Bacteriocins in *in vitro* Studies

*In vitro* antimicrobial activity assays are the first step to evaluate the biological capacity of bacteriocins against clinically relevant bacterial pathogens (Ansari et al., 2018; Peng et al., 2019). However, if the conditions used in the *in vitro* models are not adequate to assess a specific effect, then the probabilities of success in the *in vivo* models will be very low (Blay et al., 2007; Umu et al., 2016). Fortunately, many bacteriocins have been successfully tested using *in vitro* assays against relevant bacterial pathogens (including MDR pathogens) (Cui et al., 2012; Gabrielsen et al., 2014; Perez et al., 2014; Newstead et al., 2020). Some examples include bacteriocin AS-48, which is active against reference and clinical strains of *M. tuberculosis* (Aguilar-Pérez et al., 2018). Pentocin JL-1 has also been demonstrated to have antibacterial activity against Gram-positive and Gram-negative bacteria, particularly MDR *S. aureus* (Jiang et al., 2017). Other examples, such as the novel entianin, which has activity against MRSA (ATCC 43300) and vancomycin-resistant *Enterococcus faecalis* (ATCC 51299) strains (Fuchs et al., 2011), and bacteriocins klebicins have been demonstrated to be active against MDR and carbapenem-resistant *Klebsiella* species (Denkovskienė et al., 2019). Moreover, novel enterocins DD28 and DD93 showed anti-staphylococcal activity in MRSA (Al Atya et al., 2016). It is important to mention that many current reports on the study of bacteriocins are focused on pathogens considered by WHO as a priority (World Health Organization, 2017) such as carbapenem-resistant *E. coli* or *K. pneumoniae* (Chen et al., 2019), vancomycin-resistant, and MDR *E. faecium* (Phumisantiphong et al., 2017).

**FIGURE 1:**
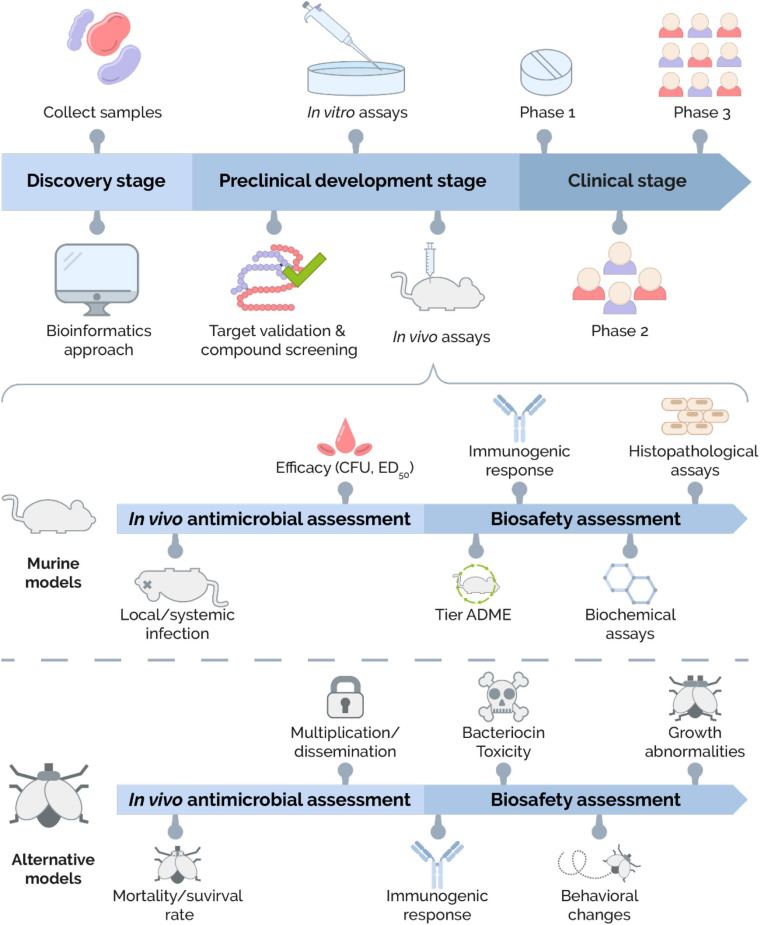
Overview of the bacteriocin development process. The bacteriocin development process is divided into three big stages: **Discovery**, **Preclinical development,** and **Clinical development.** In the **Discovery stage**, two main approaches for bacteriocins are identified. The traditional approach consists of collecting environmental samples to isolate bacteriocin-producers. On the other hand, bacteriocins can be obtained by designing and analyzing databases using a bioinformatic approach. Next, the **Preclinical development stage** is divided into three subcategories: target validation and compound screening, *in vitro* assays, and *in vivo* assays. The first subcategory focuses on screening, structure–function analysis, and characterization of bacteriocins. The second subcategory’s main goal is to demonstrate the antimicrobial activity and cytotoxicity effects by ***in vitro* assays**. The third subcategory includes the ***in vivo* assays**. The *in vivo* antimicrobial activity and biosafety assessment of bacteriocins can be carried out using murine and alternative models. The *in vivo* antimicrobial assessment in murine models includes the use of local and systemic infection models in rodents and the evaluation of efficacy. The biosafety assessment includes evaluating various parameters, such as pharmacokinetics profile (ADME), immunogenic response, and biochemical and histopathological analysis. On the other hand, the *in vivo* antimicrobial assessment in alternatives models (e.g., fruit fly, zebrafish, roundworm, greater wax moth, or brine shrimp) allows determining the mortality/survival rates and the ability of bacteriocin to block multiplication/dissemination of pathogenic agent. Biological parameters such as the immunogenic response, bacteriocin toxicity, behavioral changes, and growth abnormalities are evaluated during the biosafety evaluation. Once bacteriocin has shown to be effective and safe in *in vivo* models, it advances to the **Clinical development stage** where its dose, efficacy, and side effects are evaluated through different phases (Phases 1–3) until its approval and commercialization.

As expected, bacteriocins have an outstanding record to kill or reduce pathogens and drug-resistant pathogens during *in vitro* assessments (Fuchs et al., 2011; Cui et al., 2012; Gabrielsen et al., 2014; Ishibashi et al., 2014; Al Atya et al., 2016; Jiang et al., 2017; Aguilar-Pérez et al., 2018; Ansari et al., 2018; Denkovskienė et al., 2019; Peng et al., 2019; Newstead et al., 2020). Conventionally, bacteriocins display a non-toxic behavior at *in vitro* assays (Cebrián et al., 2019). Thus, the promising results obtained after *in vitro* assays must be extrapolated into *in vivo* assays (Kokai-Kun et al., 2003). Furthermore, at this stage, internal factors of the host (pharmacokinetic parameters) should be considered (Meade et al., 2020) as well as the potential bacteriocin-induced toxicity (Gupta et al., 2014). Therefore, the use of animal models is mandatory in the development of a new therapeutic agent and the successful results obtained from these experiments are essential to close the translational gap to the clinic (Denayer et al., 2014). The description of the preclinical drug discovery and development process is shown in Figure 1.

## *In vivo* Assessment of Bacteriocins

There are many animal models used for screening drugs or chemical compounds in preclinical testing (pharmacological bioassays) (Zwierzyna and Overington, 2017) and specific toxicity studies (toxicological bioassays) (Creton et al., 2010). Rodents (rats and mice) are the most frequently used animal species. Other animal species, less frequently used, are guinea pigs, rabbits, and hamsters (Badyal and Desai, 2014). Invertebrate models can be used to assess many biological activities, and these include *Drosophila melanogaster* (fruit fly) and *Caenorhabditis elegans* (a nematode worm) (Badyal and Desai, 2014). Finally, zebrafish is the most used rapidly developing vertebrate, and it has proven to be an excellent model for toxicity testing (Segner et al., 2003).

### *In vivo* Assessment of Bacteriocins in Murine Models

In terms of genomics, the strategies for cloning, gene knockout, and gene or genome modifications are very well-described. Genes are very well-conserved between mice and humans since they share 90% of their total genes, and they are also available to develop spontaneous mutations. In terms of biology, mice are small and easy to handle; they can be easily transported and raised in a laboratory. Gestation times of mice are relatively short, and a large number of offspring can be obtained for *in vivo* purposes (Masopust et al., 2017). Therefore, mice are the best-known animal model for *in vivo* bacteriocin efficacy studies.

As mentioned earlier, the ability of bacteriocins to kill or inhibit pathogenic bacteria *in vitro* has been well documented. Bacteriocins represent one of the most studied microbial defense systems (Cavera et al., 2015). They may facilitate the introduction of a producer into an established niche, directly inhibit the invasion of competing strains or pathogens, or modulate the composition of the microbiota and influence the host immune system (Dobson et al., 2012). Therefore, understanding that bacteriocins may function in several ways, studies involving direct correlations between *in vitro* efficacy and *in vivo* protection are needed.

To date, bacteriocins have showed a promising efficacy as antibiotic alternatives in *in vivo* studies. Bacteriocin application focuses in the administration at the site of the infection or susceptible areas, evading an immune response and maintaining the stability of the bacteriocin (Arthur et al., 2014). Therefore, selection of optimal bacteriocin and delivery systems has been complicated. Two factors seem to play an important role in *in vivo* efficacy of bacteriocins: pharmacokinetic parameters and route of administration.

First, pharmacokinetic parameters (e.g., bioavailability, stability, solubility in physiological conditions, and susceptibility to enzymatic proteolysis in bloodstream) are important determinants of the efficacy of bacteriocins (Soltani et al., 2021). Bacteriocins administered orally are exposed to the hostile environment (enzymatic and pH degradation) in the gastrointestinal tract; they are highly susceptible to degradation once they reach the small intestine. On the other hand, parenteral administration may offer some means of avoiding proteolytic degradation of bacteriocins in the gastrointestinal tract. Nevertheless, the efficacy may be reduced since bacteriocins will be in contact with proteases involved in hemostasis and fibrinolysis in the bloodstream (Meade et al., 2020). To reduce or avoid these problems, bacteriocins can be engineered to be less susceptible to proteolytic degradation by changing D-amino acids (Soltani et al., 2021). Also, nanotechnology seems to be a valuable strategy to improve the physicochemical properties of the bacteriocins (Farokhzad and Langer, 2009). The nano-encapsulation [e.g., lipid-based nanoparticles (nanoliposomes and solid lipid nanoparticles), carbohydrate-based nanoparticles (chitosan/alginate and phytoglycogen nanoparticles), and conjugation with nanosized metal] of bacteriocins could protect them from enzymatic degradation, hence increasing their stability for longer periods (Fahim et al., 2016).

Second, the route of administration determines the onset and the duration of the pharmacological effect, the efficacy, and the adverse effects of drugs. The main routes of bacteriocin administration, such as intranasal (McCaughey et al., 2016a, b), intragastric (Wongsen et al., 2019), intraperitoneal (Piper et al., 2012; Sahoo et al., 2017), subcutaneous (Kers et al., 2018a, b; Pulse et al., 2019), and topical (Van Staden et al., 2016; Cebrián et al., 2019) have demonstrated excellent efficacy in murine models. However, the efficacy of the different routes of administration has not been directly compared and likely depends on the pathogen targeted (Lohans and Vederas, 2012). In the same way, *in vivo* toxicity testing has been conducted to identify possible adverse effects resulting from exposure to bacteriocins. As described below, many studies have been done to investigate toxicity and biosafety of bacteriocins using different type of applications, such as oral, intraperitoneal, nasal, and topical. In particular, topical application of bacteriocins has been reported to be successfully tested for skin infection with no toxicity effects. For example, the circular bacteriocin AS-48 was evaluated and the results indicated that this bacteriocin did not induce skin sensitization or cause allergic contact dermatitis (Cebrián et al., 2019). Topical formulation with two broad-spectrum bacteriocins: garvicin KS and micrococcin P1 was used in a murine skin infection model. The formulation had a significant antibacterial effect and animals showed no changes of behavior or obvious toxic effects (Ovchinnikov et al., 2020). Therefore, there is a growing interest for the study of the therapeutic properties and side effects of bacteriocins using *in vivo* systems (Abanoz and Kunduhoglu, 2018; Bagci et al., 2019; Iseppi et al., 2019; Lynch et al., 2019; Lajis, 2020; Meade et al., 2020).

Latest publications on *in vivo* assessment of bacteriocins using murine models contain as minimum parameters the measurement of the antimicrobial activity of bacteriocin on the animal model, the assessment of immunogenic response, biochemical analysis, and histopathological analysis. To support this paragraph, we have summarized some of the relevant bacteriocins with their *in vivo* antimicrobial (Table 1) and toxicity and biosafety (Table 2) activities in murine models over the last 20 years. In this context, we included bacteriocin studies that have measured their effects in murine models using purified or partially purified bacteriocins (including naturally synthesized bacteriocins by native and heterologous producers and chemically synthetized bacteriocins), bioengineering bacteriocins, or the bacteriocin-producer microorganism directly in the host.

**TABLE 1:** *In vivo* antimicrobial assessment of bacteriocins using murine models.

**Bacteriocin**	**Producer**	**Target**	**Host**	**Administration route**	**Antimicrobial activity**	**References**
***In vivo* assessment of purified or partially purified bacteriocins in murine models**						
**Naturally synthetized bacteriocins by native producers**						
Mersacidin	*Bacillus* sp. HIL Y-85 54728	MRSA strain 99308	Female BALB/cA mice	Nasal	MRSA was absent in nasal cavity after treatment. Serum levels of IL-1β (inflammatory cytokine for innate immunity) and TNFα (master regulator of inflammatory response) were decreased.	Kruszewska et al., 2004
Mutacin B-Ny266	*S. mutans* Ny266	MSSA Strain	Mice	Intraperitoneal	Survival rate of infected mice with low and high doses of MSSA was 30 and 0%, respectively. Survival rate in infected mice with low and high doses of MSSA treated with B-Ny266 at 1, 3, and 10 mg/kg was 100%	Mota-Meira et al., 2005
Nisin, clausin, AmyA	*B. amyloliquefaciens* (only AmyA)	*S. aureus* Xen 36	Adult female nude mice	Skin	All antimicrobial treatments (CPVA, mupirocin, nisin, clausin, and AmyA) gradually reduced the size of wound skin infections with *S. aureus* after 7 days, although clausin- and nisin-treated wounds were smaller than CPVA-treated wounds.	Van Staden et al., 2016
Penisin	*Paenibacillus* sp. Strain A3	MRSA Strain	Male BALB/c mice	Intraperitoneal	Penisin was significantly effective at 80 and 100 mg/kg, as MRSA load decreased to 91 from 96% in mice, respectively. Survival rate in penisin-treated infected and untreated mice was 88 and 0% after day 4, respectively.	Baindara et al., 2016
AS-48	*E. faecalis strain* UGRA10	*T. cruzi* Arequipa strain	Female BALB/c mice	Intraperitoneal	55% of organs/tissues were parasite-free in mice treated with AS-48 at 1 mg/kg in both acute and chronic infection. 33 and 55% or organs were free of parasites after treatment with benznidazole at 100 mg/kg, respectively.	Martín-Escolano et al., 2020
**Naturally synthetized bacteriocins by heterologous producers**						
Lacticin 3147	*L. lactis* subsp. *cremoris* MG1363	*S. aureus* Xen 29	Female BALB/c mice	Intraperitoneal	Prevented the systemic spread of *S. aureus.* Microbial levels were decreased in the thoracic, abdominal cavity, and spleen but increased in liver and remained the same in kidney.	Piper et al., 2012
Pyocins S2, S5, AP41, and L1	*E. coli BL21(DE3)* pLysS	*P. aeruginosa* P8	Female C57/BL6 mice	Intranasally	Mice were previously infected with P8 and treated later with S2, S5, AP41, or L1. All pyocin-treated mice survived to end point (24 h post infection). S5 had the highest efficacy because no P8 was recovered in any S5-treated infected mice. All the other pyocins reduce the bacterial load by 4-log units.	McCaughey et al., 2016b
Pyocin SD2	*E. coli BL21(DE3)* pLysS	*P. aeruginosa* PAO1	Female C57/BL6 mice	Intranasally	Pyocin SD2-treated mice previously infected with PAO1 had no signs of illness and survived to end point (24 h post infection) and low counts of PAO1 were recovered from lungs (5 CFU/lung). Untreated infected mice were culled at 6 h due to severity of illness and high counts of PAO1 (10^5^ CFU/lung)	McCaughey et al., 2016a
Plantaricin E/F	*L. lactis* NZ3900	*E*. *coli* EPEC K1.1	ddY male mice	Oral	EPEC K1.1 was orally given to mice at 10^8^ CFU/ml. Then, plantaricin E and F were given at different dosages for 7 days. Leukocyte, hematocrit (due to diarrhea), and hemoglobin levels (due to damage) were increased, and erythrocyte numbers lowered during infection. After treatment with plantaricins, mice improved their healthy. Plantaricin E at 250 and 500 mg/kg and Plantaricin F at 500 mg/kg reduced inflammatory in mice as indicator of infection.	Hanny et al., 2019
**Chemically synthetized bacteriocins**						
Lysostaphin	Chemical Synthetized	MRSA (MBT 5040 and 12/12 strains), MSSA (Newman, ATCC 49521, ATCC 12605) and mupirocin-resistant (SA 3865 MupR)	Female cotton rats and female ICR mice	Nasal	Nasal colonization by *S. aureus* was eradicated at 93% in cotton rats with a single dosage of lysostaphin cream. Also, two methicillin-susceptible strains (ATCC 49521 and ATCC 12605), MRSA strain 12/12, and mupirocin-resistant SA 3865 MupR were eradicated. No antibacterial effect was observed with nisin cream at 5% (positive control).	Kokai-Kun et al., 2003
Epidermicin NI01	Chemical Synthetized	MRSA ATCC 43300	Female Cotton rats	Nasal	Untreated infected rats had mean values 3.79 log_10_ CFU/nares. MRSA-infected rats treated with NI01 at 0.8% had mean values 0.78 log_10_ CFU/nares.	Halliwell et al., 2017
***In vivo* assessment of bioengineered bacteriocins in murine models**						
Nisin A and Nisin V	*L. lactis* NZ9700 and *L. lactis* NZ9800nisA:M21V	*L. monocytogenes* EGDe	Female BALB/c mice	Intraperitoneal	Bioimaging of mice was used to quantify the bioluminescent bacteria (NZ9800NISA:M21V) in organs. Nisin V exerted a better antibacterial activity in the liver and spleen than nisin A	Campion et al., 2013
OG253	*S. mutans* 152 producing OG253 (Phe1Ile)	*C. difficile* UNT103-1	Male Golden Syrian hamsters	Subcutaneous	Survival rate for OG253-treated challenged mice were 100% while vancomycin-treated mice were 33% after 21 days. All untreated challenged mice died by day 9.	Kers et al., 2018b
OG716 and OG718	*S. mutans* 152 producing OG716 and *S. mutans* 152 producing OG718	*C. difficile* UNT103-1	Male Golden Syrian hamsters	Subcutaneous	Survival rate for OG716-treated challenged mice were 100% and vancomycin-treated mice were 83% after treatment. Control challenged mice were dead before day 5.	Kers et al., 2018a
OG716 and OG718	*S. mutans* 152 producing OG716 and *S. mutans* 152 producing OG718	*C. difficile* UNT103-1	Male Golden Syrian hamsters	Subcutaneous	*C. difficile* CFU levels in untreated challenged hamster cecum were 6.66 log_10_ CFU/ml. OG716-treated mice at 105 or 180 mg/kg body weight per day had values between <2.4 and 2.51 log_10_ CFU/ml, respectively. OG718-treated mice at 180 mg/kg BW per day had values of 3.18 log_10_ CFU/ml.	Pulse et al., 2019
***In vivo* assessment of bacteriocin-producer directly in murine models**						
Bacterial dose	*Lb. salivarius* UCC118	*L. monocytogenes* EGDe	A/J mice	Oral	*L. monocytogenes* log CFU/g levels in liver and spleen for untreated mice were 4 and 5, respectively. UCC118-treated mice had significantly lower listerial CFU levels.	Corr et al., 2007
Bacterial dose	*E. mundtii* ST4SA and *L. plantarum* 423	*S. enterica* serovar Typhimurium	Male Wistar rats	Oral	Rats were daily administered with *L. plantarum* 423 and *E. mundtii* ST4S for 7 days and then challenged with *S. enterica.* Rats gained weight (alone or combination), suggesting that bacteriocin-producers prevented the system spread of *S. enterica*.	Dicks and ten Doeschate, 2010
Bacterial dose	*E. faecium* KH24	*S. enteritidis*	Swiss Albino male mice	Oral	Mice previously fed with KH24 showed a rise in weight and 1 log CFU/g decrease in *S. enteritidis* in the small and large intestine. Neither salmonella nor enterococcal count was observed in liver and spleen.	Bhardwaj et al., 2010
Bacterial dose	*L. plantarum* B7	*S. typhimurium* ATCC 13311	Male albino mice	Oral	*S. typhimurium* levels in feces of untreated infected mice (7.42 ± 0.05 logCFU/g) were higher than B7-treated infected mice (8.86 ± 0.02 logCFU/g). Serum levels of TNF-a, IL-6, and CXCL1 were higher in untreated infected mice than in pretreated infected mice.	Wongsen et al., 2019

**TABLE 2:** *In vivo* toxicity and biosafety assessment of bacteriocins using murine models.

**Bacteriocin**	**Producer**	**Host**	**Toxicity and biosafety assessment**	**References**
***In vivo* assessment of purified or partially purified bacteriocins in murine models**				
**Naturally synthetized bacteriocins by native producers**				
Mutacin B-Ny266	*S. mutans* Ny266	Mice	No toxicity was recorded of B-Ny266 at 10 mg/kg	Mota-Meira et al., 2005
Nisin, clausin, AmyA	*B. amyloliquefaciens* (only AmyA)	Mice	CPVA, mupirocin, nisin, clausin, and AmyA gradually reduced the size of non-infected wounds after 7 days. No toxicity assessment was displayed.	Van Staden et al., 2016
TSU4	*L. animalis* TSU4	Male BALB/c mice	TSU4 over 200 mg/kg body weight was safe enough. No significant impact of bacteriocin on the kidney and liver after sub-chronic toxicity test.	Sahoo et al., 2017
AS-48	*E. faecalis* strain UGRA10	Female BALB/c mice	Serum biochemical measurements were performed to evaluate *in vivo* toxicity in mice (5 mg/kg). AS-48 made changes in biochemical measurements. No mice died or lost more than 5% body weight. After 7 days, mice returned to normal levels.	Cebrián et al., 2019
AS-48	*E. faecalis strain* UGRA10	Female BALB/c mice	None of the treated mice died and lost more than 10% body weight after treatment.	Martín-Escolano et al., 2020
**Naturally synthetized bacteriocins by heterologous producers**				
Pyocins S2, S5, AP41, and L1	*E. coli BL21(DE3)* pLysS	Female C57/BL6 mice	Pyocins S2, S5, and L1 except AP41 were stables in the lung and do not cause inflammation or tissue damage in murine lung. AP41 was presumably degraded in lungs.	McCaughey et al., 2016b
Plantaricin E/F	*L. lactis* NZ3900	Male ddY mice	Bacteriocin E or F at concentrations ranging from 50, 100, 1000, and 5000 mg/kg body weight had no mortality in mice. Hematological and biochemical parameters displayed normal levels and histopathology shows normal liver and kidney cells. The leukocyte, hematocrit, and hemoglobin levels in mice were improved after bacteriocin treatment; also, the malondialdehyde (MDA) indicator levels were reduced.	Hanny et al., 2019
**Chemically synthetized bacteriocins**				
Epidermicin NI01	Chemical synthetized	Female Cotton rats	Histology studies of the nasal cavities demonstrated mild to a marked epithelial abnormality with a decreasing gradient of severity from the anterior to posterior regions of the mice nasal cavities in epidermicin NI01 at 0.2%. No cytotoxic activity, necrosis, or presence of blood was noted.	Halliwell et al., 2017
***In vivo* assessment of bioengineered bacteriocins in murine models**				
OG716 and OG718	*S. mutans* 152 producing OG716 and *S. mutans* 152 producing OG718	Male Golden Syrian hamsters	OG716 and OG718 were administered at doses of 180 mg/kg body weight (mg/kg BW) of hamsters challenged with *C. difficile* protected to the rodent since survival rate was around 80% after 22 days of treatment	Pulse et al., 2019
***In vivo* assessment of bacteriocin-producer directly in murine models**				
Bacterial dose	*E. mundtii* ST4SA and *L. plantarum* 423	Male Wistar rats	Endotoxin levels were lowered in rats that received *L. plantarum* 423 and *E. mundtii* ST4SA. No signs of splenomegaly or hepatomegaly were observed in tissue samples taken from the liver and spleen, and no abnormal morphological changes were observed in the epithelium of the ileum and colon.	Dicks and ten Doeschate, 2010
Bacterial dose	*E. faecium* KH24	Swiss Albino male mice	Total fecal enterococcal, *Lactobacilli*, and coliform counts (log CFU/g fecal sample) were higher in mice fed with bacteriocin-producer strains than non-bacteriocin-producer strains.	Bhardwaj et al., 2010
Bacterial dose	*L. acidophilus* JCM1132 (bacteriocin-producer) and CCFM720 (non-bacteriocin-producer)	C57BL/6J male mice	JCM1132 strain (bacteriocin-producer) reduced the proinflammatory cytokine IL-6 in mice. CCFM720 strain (non-bacteriocin-producer) decreased concentration of anti-inflammatory factor IL-10. Both strains showed low immunogenicity. No significant immune response was recorded. CCFM720 favored the prevention of metabolic diseases. JCM1132 showed weak inflammatory response in comparison to CCFM720-treated mice.	Wang et al., 2020

#### *In vivo* Assessment of Purified or Partially Purified Bacteriocins in Murine Models

For categorization purposes, in this section, we only include studies of bacteriocins that were applied purely or partially purified in murine models. We here consider bacteriocins that were chemically synthesized and those that were naturally synthesized from a native bacteriocin-producer or by a heterologous bacteriocin-producer.

##### Naturally synthetized bacteriocins by native producers

This group is characteristic since it is a straightforward system that produces bacteriocins. Native bacteriocin-producers usually excrete bacteriocins by dedicated membrane-associated ATP-binding cassette (ABC) transporters or by the general secretion (*sec*) pathway of the cell (Munoz et al., 2011).

Mersacidin is a lantibiotic-type bacteriocin that was isolated and purified from *Bacillus* spp. HIL Y-85 54728. It has been tested in female BALB/cA mice infected by *S. aureus* 99308. Results showed a decrease in the inflammatory response of the host (Kruszewska et al., 2004; Parameswaran and Patial, 2010; Kaneko et al., 2019). Mutacin B-Ny266 is naturally produced from *Streptococcus mutans* Ny266. Its antibacterial effect was proved in mice infected with methicillin-susceptible *S. aureus* (MSSA) strain. Moreover, no mortality was observed in mutacin B-Ny266-treated mice (Mota-Meira et al., 2005). Another bacteriocin is nisin; it has been tested along with clausin and the two components (α- and β-peptides) bacteriocin amyloliquecidin (AmyA) from *Bacillus amyloliquefaciens* against a bioluminescent strain of *S. aureus* Xen 36 in adult female nude mice in a wound skin infection model. Interestingly, all antimicrobial treatments reduced the bacterial load after 7 days of treatment (Van Staden et al., 2016). Penisin, from *Paenibacillus* sp. Strain A3, was used to effectively protect mice from a MRSA infection. Penisin-treated infected mice had a significant higher survival rate than untreated infected mice (Baindara et al., 2016). TSU4 is a bacteriocin recovered from *Lactobacillus animalis* TSU4. This bacteriocin was used in BALB/c mice to evaluate the acute and sub-chronic toxicity tests. Biochemical and histopathological analysis was performed. The bacteriocin demonstrated to be safe in a sub-chronic toxicity test. No antimicrobial *in vivo* test was performed (Sahoo et al., 2017). Finally, AS-48 bacteriocin is produced by *E. faecalis* strain UGRA10. The immunogenic response and biochemical and histopathological effects were analyzed in BALB/c mice (Cebrián et al., 2019). Later, its activity as antiprotozoal peptide was tested in BALB/c mice challenged with *Trypanosoma cruzi* strain Arequipa (Chaga’s disease etiological agent). Results demonstrated that this bacteriocin reduced the acute infection in mice (Martín-Escolano et al., 2020).

##### Naturally synthetized bacteriocins by heterologous producers

Occasionally, the internal mechanisms of the native bacteriocin-producers to produce and excrete the bacteriocins are not enough. The objective to produce the bacteriocin in a heterologous expression system is to increase the bacteriocin production yield from native producers by facilitating the control of gene expression or increasing the production levels (Mesa-Pereira et al., 2018).

Lacticin 3147 is a two-peptide bacteriocin that is heterologous-produced in the recombinant strain *Lactococcus lactis* subsp. *cremoris* MG1363. This bacteriocin has been tested in an animal model, and the results have showed that it was able to reduce *in vivo* infection with *S. aureus* Xen 29 in mice (Piper et al., 2012). A very well-known example of bacteriocins from Gram-negative bacteria are pyocins, which are believed to be produced in 90% of *P. aeruginosa* strains (Michel-Briand and Baysse, 2002). The heterologous-produced pyocins S2, S5, AP41, and L1 were used to study their protective function in an acute *P. aeruginosa* lung infection in C57/BL6 mice. Among all the pyocins, S5 was the best because no pathogenic bacteria were recovered from any of the S5 treated mice. The remaining pyocins were able to reduce the bacterial count in their respective treated mice (McCaughey et al., 2016b). Shortly after, from the heterologous-produced pyocins SD1, SD2, and SD3, pyocin SD2 exerted the best performance among the other pyocins when it was tested in previously challenged C57/BL6 mice with *P. aeruginosa* PAO1. Treated mice were able to survive, and no signs of illness were reported (McCaughey et al., 2016a). On the other hand, plantaricin E/F are two bacteriocins (plantaricin E and F) that have been heterologously produced in *L. lactis* NZ3900. The *in vivo* effects of both plantaricins were tested independently in a murine model infection. The favorable results obtained in antibacterial and toxicological tests suggest that plantaricin E or F are unharmful compounds that can be considered as a strong antibiotic candidate (Hanny et al., 2019).

##### Chemically synthetized bacteriocins

Chemically synthesized bacteriocins are bacteriocins that were previously reported on non-modified bacteriocin-producer strains, and their antimicrobial effect have been measured on *in vivo* assays, but they have been synthesized chemically; some examples are lysostaphin and epidermicin NI01. First, lysostaphin was formulated on a petroleum-based cream, and it was able to eradicate *S. aureus* strain MBT 5040 in cotton rats after one single application (Kokai-Kun et al., 2003). Second, the efficacy of epidermicin NI01, for eradicating the nasal burden of MRSA strain ATCC 43300 in a cotton rat model, was carried out. Results showed that a single dose of topical epidermicin NI01 was effective in eradication of MRSA from the nares of rats (Halliwell et al., 2017).

#### *In vivo* Assessment of Bioengineered Bacteriocins in Murine Models

Bioengineered bacteriocins are a group of bacteriocins whose characteristics have been modified to upgrade their properties. These modifications consist of the generation of novel bacteriocin variants that enhance the antimicrobial activity or expand the antibacterial spectrum and anti-biofilm efficacy or improve their physicochemical properties (Field et al., 2018). Some examples of this type of bacteriocins are described below.

*In vivo* activity of nisin A and nisin V against *Listeria monocytogenes* was evaluated in mice. Nisin V (a modified version of Nisin A) was more effective than Nisin A to controlling infection (Campion et al., 2013). *Mutacin* 1140 (MU1140) is a lantibiotic produced by *S. mutans*. A study to identify a lead compound for the treatment of *Clostridium difficile*-associated diarrhea was carried out. The variant OG253 emerged as the lead compound based on superior *in vivo* efficacy along with an apparent lack of relapse in a hamster model of infection (Kers et al., 2018b). *In vivo* testing of another MU1140-derived variant (OG716) conferred 100% survival and no relapse at 3 weeks post *C. difficile* infection (Kers et al., 2018a). Also, the effect of OG716 is determined using an *in vivo* hamster model of *C. difficile*-associated disease. Results demonstrated that OG716 was an excellent compound to treat *C. difficile* enteritis in hamsters (Kers et al., 2018a).

#### *In vivo* Assessment of Bacteriocin-Producer Directly in Murine Models

Bacteriocin-producers act similarly to probiotics because both can be consumed and exert a health benefit to the host. As long as they stay in the host, they may act as colonizing peptides, killing peptides, or serve as signaling peptides (signaling other bacteria or the immune system) (Dobson et al., 2012). It has been shown that bacteriocin production by bacteriocin-producers in the gut of the host can modulate the niche competition by preventing the intestinal colonization of MDR bacteria without disturbing the natural microbiota, therefore limiting the infection (Kommineni et al., 2015; Hegarty et al., 2016).

A study demonstrated that *Lactobacillus salivarius* UCC118 (a sequenced and genetically tractable probiotic strain of human origin) significantly protected mice against infection with the invasive foodborne pathogen *L. monocytogenes* (Corr et al., 2007). In other study, rats were administered daily with *Lactobacillus plantarum* 423 and *Enterococcus mundtii* ST4SA. Then, they were challenged by infection with *Salmonella enterica* serovar *Typhimurium*. Results showed that *L. plantarum* 423 was more effective than *E. mundtii* ST4SA (Dicks and ten Doeschate, 2010). Bhardwaj et al. (2010) performed the safety assessment and evaluation of probiotic potential of bacteriocinogenic *E. faecium* KH 24 strain using an *in vivo* model. Mice were challenged with *Salmonella enteritidis* MTCC 3291 and fed with *E. faecium* KH 24 strain. Results showed beneficial intestinal results (decreased counts of bacteria and coliform, and enhanced growth of lactobacilli) (Bhardwaj et al., 2010). The protective effects of *L. plantarum* B7 on diarrhea in mice induced by *Salmonella typhimurium* ATCC 13311 was evaluated. Results demonstrated that *L. plantarum* B7 could inhibit growth of *S. typhimurium*, decrease levels of proinflammatory cytokines, and attenuate symptoms of diarrhea induced in mice by *S. typhimurium* (Wongsen et al., 2019). Another study evaluated the effects of the bacteriocin-producing *Lactobacillus acidophilus* strain JCM1132 and its non-producing spontaneous mutant, *L. acidophilus* CCFM720, on the physiological statuses and gut microbiota of healthy mice. The results showed that both strains can have different effects on the host such as prevention of metabolic diseases and reduced inflammatory response (Wang et al., 2020).

### *In vivo* Assessment of Bacteriocins in Alternative Models

The use of alternative models has gained great popularity among the scientific community since these models are simple, fast, and cheaper than current murine models (Apidianakis and Rahme, 2009; Jennings, 2011; OECD, 2013; Rajabi et al., 2015; Son et al., 2016; Herndon et al., 2017; Ignasiak and Maxwell, 2017; Jackson et al., 2017; Tseng et al., 2019). As we previously mentioned, some of these alternative models include *D. melanogaster* (fruit fly), *Danio rerio* (zebrafish embryos), *C. elegans* (roundworm larvae), *Galleria mellonella* (greater wax moth larvae), and *Artemia salina* (brine shrimp larvae) (Freires et al., 2017). These alternative models allow evaluating bacteriocins and their potential effects on a living organism (such as antimicrobial activity, antibiofilm effect, immunogenic response, and toxicity) (Niu et al., 2014; Thomsen et al., 2016; Hunt, 2017; Cutuli et al., 2019; Yi et al., 2019). Also, murine models and alternative models do not share the same ethical considerations since the first ones have more restrictions when it comes to conducting experiments (Baertschi and Gyger, 2011; Desalermos et al., 2011; Jennings, 2011; Hamidi et al., 2014; Simonetti et al., 2016; Tsai et al., 2016; Ignasiak and Maxwell, 2017).

A compilation of bacteriocin studies with their *in vivo* antimicrobial activity (Table 3) and/or toxicity and biosafety (Table 4) activity using alternative models over the last 10 years is shown below. In this context, we included studies that have measured bacteriocin effects in alternative models using purified or partially purified bacteriocins (including naturally synthetized bacteriocins by native and heterologous producers and chemically synthesized bacteriocins) or CFS with bacteriocin-like substance or with a bacteriocin-producer directly in the host.

**TABLE 3:** *In vivo* antimicrobial assessment of bacteriocins using alternative models.

**Bacteriocin**	**Producer**	**Target**	**Host (Linnaeus/common name)**	**Antimicrobial activity**	**References**
**Naturally synthetized bacteriocins by native producers**					
NAI-107	ND	*S. aureus* USA300 (MRSA)	*D. melanogaster* (fruit fly)	One dosage of NAI-107 (100 × MIC) rescued 50–60% of MRSA-infected adult flies after 96 h.	Thomsen et al., 2016
Lichenicin 146	*B. licheniformis* strain 146	*S. aureus* RF122	*C. elegans* (roundworm)	Survival rate of untreated infected nematodes was less than 10%, but treated nematodes had 74%.	Son et al., 2016
**Naturally synthetized bacteriocins by heterologous producers**					
Pyocin S2	*E. coli*	*P. aeruginosa* YHP14	*G. mellonella* (greater wax moth)	Untreated infected larvae died after 12–14 h. YHP14 level counts in subjects were 5 × 10^8^ and 1 × 10^9^ CFU at death time. Treated larvae had 100% survival rate after 72 h.	Smith et al., 2012
Peocin	*E. coli* BL21	*A. hydrophila*	*D. rerio* (zebrafish)	Survival rates of infected zebrafish embryos were 63.3 ± 7.63 and 71.67 ± 2.88% when 1 and 5 μg, respectively, were applied.	Tseng et al., 2019
**Chemically synthetized bacteriocin**					
Epidermicin NI01	Chemically synthetized	MRSA and MSSA ATCC 11195	*G. mellonella* (greater wax moth)	Epidermicin at 200 mg/kg effectively increased the survival of infected larvae after 2 h post infection with either MRSA or MSSA.	Gibreel and Upton, 2013
**Bacteriocin-producers used directly**					
Bacterial dose	*Bacillus* sp. LT3	*V. campbellii*	*A. franciscana* (brine shrimp)	Larvae pretreated with LT3 cultures at 10^7^ cells/ml exerted a protective effect in larvae challenged with *V. campbellii*.	Niu et al., 2014
Bacterial dose	*L. fermentum* JDFM216	*S. aureus* and *E. coli* O157:H7	*C. elegans* (roundworm)	Previously colonized worms with bacteriocin-producer prolonged the lifespan of the nematodes infected against *S. aureus* and O157:H7. Uncolonized infected worms died after 10 days.	Park et al., 2018
AS-48	*E. faecalis* UGRA10	*L. garvieae*	*O. mykiss* (rainbow trout)	Trout challenged with *L. garvieae* and UGRA10 administered in diet 30 days before infection had a cumulative survival rate of 50% compared with 0% for control fish.	Baños et al., 2019
Bacterial dose	*P. pentosaceus* SL001	*A. hydrophila*	*C*tenopharyngodon *idella* (grass carp)	Cumulative mortality rates in untreated grass (only with *A. hydrophila*) was among 90%, but adapted grass was 51.7% (SL001 plus *A. hydrophila*).	Gong et al., 2019
Bacterial dose	*C. aquaticum*	*A. hydrophila* and *S. iniae*	*D. rerio* (zebrafish)	Fish previously fed with 10^6^ CFU/g *C. aquaticum* had survival rates 49.9 ± 3.88% when infected with *A. hydrophila* or 53.3 ± 7.69% when infected with *S. iniae*. Unprotected fish and infected with *A. hydrophila* or *S. iniae* had survival rates among 28.8 ± 7.69 or 17.7 ± 3.85%, respectively	Yi et al., 2019

**TABLE 4:** *In vivo* toxicity and biosafety assessment of bacteriocins using alternative models.

**Bacteriocin**	**Producer**	**Host (Linnaeus/common name)**	**Toxicity and biosafety**	**References**
**Naturally synthetized bacteriocins by native producers**				
Unnamed bacteriocin	*L. lactis*	*A. salina* (brine shrimp)	LC_50_ value was 21.54 μg/ml. The immune response was not measured.	Rajaram et al., 2010
LR14	*L. plantarum* LR/14	*D. melanogaster* (fruit fly)	LC_50_ value was 10 mg/ml. 100% lethality was observed at 20 mg/ml. No significant mortality was reported at 1, 2, and 5 mg/ml.	Gupta et al., 2014
NAI-107 and nisin	ND	*D. melanogaster* (fruit fly)	Immunogenic response NAI-107 and nisin (both alone) were measured by quantifying expression of *drs*, *cecA1*, and *attB*. In general, NAI-107 (100 × MIC) has shown a significantly low immunogenic response than nisin (3 × MIC). Survival rate of NAI-107-treated (alone) flies was higher than nisin-treated (alone) flies. Thus, NAI-107-treated flies exerted lower toxicity.	Thomsen et al., 2016
AS-48	*E. faecalis* UGRA10	*D. rerio* (zebrafish)	Concentrations at 0.64 and 1.39 μM were unharmful for 24 to 48 h. No damage at 3 μM in the first 24 h, but 30% of embryos were dead after 48 h post-treatment. 100% lethality was observed at 6.4 and 14 μM after 24 to 48 h post-treatment.	Cebrián et al., 2019
**Naturally synthetized bacteriocins by heterologous producers**				
Pyocin S2	*E. coli*	*G. mellonella* (greater wax moth)	Uninfected pyocin-treated moth larvae (control) had a survival rate of 100% with pyocin at 27 mg/kg.	Smith et al., 2012
Peocin	*E. coli* BL21	*D. rerio* (zebrafish)	No toxicity was recorded when 5 μg of peocin was used. However, mortality increased when dosages were over 10 and 20 μg.	Tseng et al., 2019
**Chemically synthetized bacteriocin**				
Epidermicin NI01	Chemically synthetized	*G. mellonella* (greater wax moth)	Bacteriocin suspensions at 200 mg/kg were unharmful (neither dead nor injuries) to larvae. No significant differences were found in the hemocyte density (indicator larval immune system) between control and epidermicin-treated larvae.	Gibreel and Upton, 2013
**Cell-free supernatant with bacteriocin-like substance**				
Bacteriocin	*E. hirae*	*A. salina* (brine shrimp)	No toxicity was recorded with CFS/BLIS at 10 and 100 mg/ml.	Azab et al., 2016
Bacteriocin	*L. curvatus* P99	*D. melanogaster* (fruit fly)	Concentrations lower than or equal to 42,109.5 AU/ml were unharmful (survival rate 98.9%). Concentrations greater than 50,000 AU/ml were lethal (survival rate less than 50%)	Funck et al., 2019
**Bacteriocin-producers used directly**				
Bacterial dose	*Bacillus* sp. LT3	*A. franciscana* (brine shrimp)	Innate immune response genes for melanization and coagulation (*proPO* and *tgase*) were not stimulated by the presence of LT3 strain (alone)	Niu et al., 2014
Bacterial dose	*L. fermentum* JDFM216	*C. elegans* (roundworm)	Bacteriocin-producer enhances the expression of *pmk-1* pathway in *C. elegans* and, thus, stimulates the immune response and longevity of *C. elegans.*	Park et al., 2018
AS-48	*E. faecalis* UGRA10	*O. mykiss* (rainbow trout)	UGRA10 was administrated in tanks filled with the fish and water for 15 days. No deaths or visible signs and injuries were seen. No cytotoxicity was observed in R1 cell line and trout.	Baños et al., 2019
Bacterial dose	*P. pentosaceus* SL001	*C. idella* (grass carp)	No mortality neither adverse effects were reported at high population concentrations in fish diet	Gong et al., 2019
Bacterial dose	*C. aquaticum*	*D. rerio* (zebrafish)	Zebrafish injected with the bacteriocin-producer increased in the expression of carbohydrate metabolism-related genes in the liver and innate immune-related genes were induced.	Yi et al., 2019

#### *In vivo* Assessment of Purified Bacteriocins in Alternative Models

##### Naturally synthetized bacteriocins by native producers

A fruit fly model (*D. melanogaster*) was used to evaluate the acute toxicity of antimicrobial peptide LR14. The results showed that the peptide had a dose-dependent toxicity property (Gupta et al., 2014). Another study used the same *in vivo* model to evaluate the efficacy of peptide NAI-107 as treatment in MRSA infections. The authors reported that this peptide was able to rescue adult flies from fatal infection with efficacy equivalent to that of reference antibiotic (vancomycin) (Thomsen et al., 2016). The antibacterial spectrum and cytotoxicity of a bacteriocin produced by *Lactobacillus lactis* strain in *A. salina* nauplii. The antibacterial activity of bacteriocin showed a broad range against foodborne pathogens. Also, the purified bacteriocin showed cytotoxicity in brine shrimps (Rajaram et al., 2010). Son et al. (2016) identified a novel bacteriocin produced by *Bacillus licheniformis* strain 146 (lichenicin 146) with a high *in vivo* antimicrobial activity in liquid *C. elegans*–*S. aureus* assay (Son et al., 2016).

AS-48 is a bacteriocin produced by *Enterococcus* strains. The toxicity of this bacteriocin has been evaluated in *in vivo* models. In zebrafish embryos, the AS-48 was highly toxic; however, in a murine model, no toxicity was observed (Cebrián et al., 2019).

##### Naturally synthetized bacteriocins by heterologous producers

A nonvertebrate host, the *G. mellonella* caterpillar, was used to evaluate the activity of pyocin S2 against *P. aeruginosa* YHP14 biofilms. Results showed a potent antibiofilm activity *in vivo*, representing a potential therapeutic option (Smith et al., 2012). The antimicrobial activity of peocin, a bacteriocin produced by the probiotic *Paenibacillus ehimensis* NPUST1, was demonstrated in aquatic, food spoilage, clinical, and antibiotic-resistant pathogens. For example, a significant increase in survival rates was observed in peocin-treated zebrafish after *Aeromonas hydrophila* challenge (Tseng et al., 2019).

##### Chemically synthetized bacteriocin

A study reported that epidermicin NI01 had a protective effect of *G. mellonella* larvae from infection with clinically isolated MRSA. The authors reported that epidermicin NI01 did not induce toxicity and did not trigger the larvae immune system (Gibreel and Upton, 2013).

##### *In vivo* assessment of CFS with bacteriocin-like substance in alternative models

The *in vivo* assessment of bacteriocins in alternative models has been evaluated using a CFS that contains a bacteriocin like-substance (BLIS). For example, *A. salina* brine shrimp showed no toxicity of CFS with BLIS from *Enterococcus hirae* (Azab et al., 2016). Recently, CFS from *Lactobacillus curvatus* P99 cultures showed no toxic effect in *D. melanogaster* flies (Funck et al., 2019).

#### *In vivo* Assessment of Bacteriocin-Producer Directly in Alternative Models

The evaluation of the probiotic effect of *Bacillus* sp. LT3 was performed in brine shrimp *Artemia franciscana* larvae challenged with *Vibrio campbellii* LMG 21363. *Bacillus* sp. LT3 was able to colonize the brine shrimp gastrointestinal tract and therefore increased their survival (Niu et al., 2014). A *C. elegans* model was used to evaluate the functionality of *Lactobacillus fermentum* strain JDFM216 (a novel probiotic bacterium). Interestingly, the probiotic bacterium was found to be toxic to the *C. elegans* host. Therefore, it has beneficial effects on longevity and immunity of *C. elegans* (Park et al., 2018). The effect of *Pediococcus pentosaceus* strain (SL001) in growth-related and immune-related genes was evaluated in grass carps. Results showed that the strain was able to enhance immunity and promoter growth of grass carps (Gong et al., 2019). A recent study evaluated the effect of probiotic *Chromobacterium aquaticum* on zebrafish model. Fish was challenged with *A. hydrophila* and *Streptococcus iniae* and then treated with probiotic. The probiotic-treated fish had a higher survival rate than the non-treated fish (Yi et al., 2019). Finally, the effect of *E. faecalis* UGRA10 and its bacteriocin AS-48 (multiple baths and single dose) was tested against *Lactococcus garvieae* in an *Oncorhynchus mykiss* rainbow trout fish model. Neither the strain nor its bacteriocin showed toxic effects, displaying a protective effect against *L. garvieae* infection (Baños et al., 2019).

The *in vivo* assessment models are important to evaluate bacteriocins. This review included various bacteriocins that were assessed by different *in vivo* models. The animal model must be chosen according on the expect effects for the bacteriocins. Below is summarized (Table 5) some advantages and disadvantages of murine, fruit flies, zebrafish, roundworm, greater wax moth, and brine shrimp for drug discovery trials. We included the limitations and strengths of each model as well as their scope of interest, time to reach the optimal developmental stage according to model, frequency of use in drug discovery studies, infrastructural and cost requirements for rearing, special qualifications, and ethical considerations, among others, which seem to be expected for the evaluation of new bacteriocins depending on the used model.

**TABLE 5:** Animal models as a tool to bacteriocins analysis: strengths and limitations.

**Animal model**	**Strengths**	**Limitations**	**References**
Mice	Physiology and genetics similarities to humans. Mouse genome is very similar to human. Therefore, it is a powerful tool for modeling specific genetic diseases. Extremely useful for studying complex biological systems (e.g., immune, endocrine nervous, cardiovascular, and skeletal systems). Useful for toxicity and safety assessments of new compounds. Cost-effective model because they are small, inexpensive, and easy to maintenance.	Legal and ethical considerations. Relatively large numbers of animals are needs for research. Mice models of human disease should not be utilized to supplant testing in conventional animal models.	Bryda, 2013; Morgan et al., 2013; Perlman, 2016; Andersen and Winter, 2019
*Drosophila melanogaster* (fruit fly)	Ideal for the study of genetics and development. Used to test the toxicity of chemical. 75% of the genes that cause disease in humans are also found in the fruit fly. It is relatively straightforward to mutate (disrupt or alter). Low cost to maintain in the laboratory. No ethical considerations.	Important vertebrate-specific pathogenetic factors may be ignored. Lack of an adaptive immune system and dramatically different drug effects when compared to human studies.	Jeibmann and Paulus, 2009; Pandey and Nichols, 2011; Rand et al., 2014; Mirzoyan et al., 2019
*Caenorhabditis elegans* (roundworm larvae)	Most widely used and versatile model for biological and genomic research. Used in longevity and senescence studies. Ideal for neural networks and behavior studies. Simple anatomy, optical transparency, short lifespan. Easy to work with; minimal nutritional and growth requirements.	Fewer gene homologs in mammals. Worm has two sexes (male and hermaphrodites). Down-regulation or desensitization of target genes or proteins.	Teschendorf and Link, 2009; Tissenbaum, 2015; Meneely et al., 2019
*Danio rerio* (zebrafish embryos)	70% of human genes have at least one obvious zebrafish ortholog. Used successfully to study human disease-related genes. Ideal model organism for studying early development. Drug safety testing and ecotoxicological screening. Its small size, accessibility, and transparency allow the analysis of thousands of live animals at single-cell resolution. This system is cheap and fast to develop, and it can be used by small laboratories.	Legal and ethical considerations (some countries). They require water system to maintain them. They are not as closely related to humans as mouse is (e.g., anatomy and physiology). Genes with similar sequences often have overlapping or partially redundant functions, resulting in no or subtle defects on disruption of a single gene.	Ali et al., 2011; Howe et al., 2013; Schier, 2013; Sloman et al., 2019
*Galleria mellonella* (greater wax moth larvae)	Used to study pathogenesis, virulence mechanisms, and immune response. Important tool for the preliminary screening of antimicrobial compounds. Rapid and reliable evaluation of the activity and toxicity of novel antimicrobial drugs. Larvae can survive at mammalian physiological temperature (37°C). Good correlation between toxicity in Galleria and that in rodents.	Lack of standardized procedures to use as a non-mammalian infection model. Toxicity experimental data (LD50) do not necessarily correlate to human values.	Ignasiak and Maxwell, 2017; Cutuli et al., 2019
*Artemia salina* (brine shrimp larvae)	It is a preliminary toxicity screen. Used in applied toxicology and ecotoxicity studies (high throughput cytotoxicity test of bioactive chemicals). Rapid hatching and easy accessibility of nauplii in a cost-efficient way. Easy handling under laboratory conditions.	Lack of standardized experimental conditions (temperature, salinity, aeration, light, and pH). Use the same age of *larvae* at the start of every test.	Nunes et al., 2006; Hamidi et al., 2014; Wu, 2014; Yu and Lu, 2018

## Future Trends in Bacteriocin Development and Design

Bacteriocins represent a potential drug alternative for replacing current antibiotics to treat diseases caused by resistant bacteria. According to the body of knowledge that has been developed in the field, in general, bacteriocins can retain their *in vivo* antimicrobial properties in a challenged host, while at the same time, they showed a null or reduced toxic effect. According to 2019 WHO’s Antibacterial Agent in Preclinical Development Book, 27 of 252 antimicrobial agents in preclinical revision status are considered as antimicrobial peptides. From the total antimicrobial peptides, 12 peptides are on lead optimization (LO) phase, 12 peptides are in Preclinical Candidate (PCC) phase and, three peptides are on CTA/IND-enabling studies (World Health Organization, 2019b). In an independent study, Theuretzbacher et al. (2020) identified the current global antibacterial pipeline and found that 135 of 407 preclinical projects from 314 private and public institutions were related to producing synthetic and natural antimicrobial peptides, natural products, and LpxC inhibitors, and most of these molecules are targeting Gram-negative bacteria. Therefore, bacteriocins may have a window of opportunity to face the drug-resistant bacteria crisis since the WHO is demanding research and development of new drugs to target the most wanted drug-resistant pathogens, many of them Gram-negative bacteria (World Health Organization, 2017).

Although it is a fact that the current literature for bacteriocins produced from Gram-negative bacteria is dominated by bacteriocins toward Gram-positive bacteria (Jamali et al., 2019), there is an acceptable amount of bacteriocins reported to have a strong activity against Gram-negative bacteria, including the pathogenic strains. For example, the S-type pyocin group from the *P. aeruginosa* (Ghequire and De Mot, 2014) or the microcin and colicin groups that are vastly reported for *E. coli*, and other examples in less quantity but not less relevant, are bacteriocins produced by *K. pneumoniae, Citrobacter freundii*, *Shigella boydii*, and *Serratia marcescens* (Yang et al., 2014).

The future of bacteriocins lies not only in their discovery but also in their testing for toxicity to prove their safe use in a preclinical phase as candidates for therapeutic processes. An increasing approach that can be exploited for bacteriocins is the use of alternative models to the murine model to evaluate their *in vivo* antimicrobial and/or toxicity effects. According to Freires et al. (2017), there is an increase in drug studies using alternative organisms to murine, since there was a rise of 909% in drug discovery from 1990 to 2015.

Finally, we have stated that bioengineering is an important tool along with the current technologies to discover new bacteriocins, since they can improve their antimicrobial activity or change their physicochemical properties. Moreover, new strategies are being introduced in the design of bacteriocins. Fields et al. (2020a) were the first to design the very first fully *de novo* bacteriocin by using a machine-learning approach. Fields et al. (2020b) also constructed a library of the linear peptides from the membrane-interacting region of circular bacteriocin with pore-formation dynamics by selective aminoacidic substitution. On the other hand, Acuña et al. (2012) were the first to design chimeric bacteriocins that retained the properties to kill both Gram-positive and Gram-negative bacteria. Moreover, other authors have preferred to repurpose the bacteriocins by exploiting their capability against tumor cells (Varas et al., 2020) or by exploring synergistic effects of bacteriocins while combining with renowned antibiotics (Mathur et al., 2017) or with other bacteriocins (Hanchi et al., 2017).

## Conclusion

Bacteriocins are strong candidates to be used as future therapeutic agents. Recent studies have shown the antibacterial activity of bacteriocins using *in vitro* models. Nonetheless, the next step in the development of a new bacteriocin-based therapeutic agents involves the use of animal models. The antimicrobial and/or toxic effects of the bacteriocins have been studied in murine models and the most recent alternative animal models such as fruit flies, zebrafish embryos, roundworm, greater wax moth, or the brine shrimp. These results have demonstrated that bacteriocins can exert a variety of positive responses in the host such as modification of the immunogenic response, alteration of the inflammatory response, and the reduction of biochemical and histopathological parameters related with infection. However, approximately half of the bacteriocins tested in mice (47.4%) performed antimicrobial assay, but no toxicity assays were described. On the other hand, 20% of the studies carried out in alternative models evaluated antimicrobial activity, but no toxicity was reported. These data reveal the lack of toxicity and biosafety studies of bacteriocin *in vivo* models, which are crucial to advance into clinical trials. Therefore, it is imperative to use *in vivo* models to assess the therapeutic efficacy of bacteriocins as well as their toxic effects; both successful results will lead toward clinical research phases and the development of bacteriocin-based therapeutics to treat infections caused by antimicrobial-resistant Gram-positive and Gram-negative bacteria.

## Author Contributions

DB-C, AL-B, and JM-R: conceptualization. DB-C, AL-B, JL-E, and JM-R: writing—original draft preparation and writing—review and editing. AL-B and JM-R: supervision. All authors contributed to the article and approved the submitted version.

## Conflict of Interest

The authors declare that the research was conducted in the absence of any commercial or financial relationships that could be construed as a potential conflict of interest.
